# Supplementary material for: Heat adaptation of phage T7 under an extended genetic code
Source: Virus Evol. 2021 Dec 1;7(2):veab100. doi: 10.1093/ve/veab100 (PMC8923235; doi:10.1093/ve/veab100)
Supplement: veab100_Supp [file veab100_supp.zip › Sequencing_Analysis.zip!/Sequencing_Analysis/S3/evidence/JC_SIDE_2_47.html]

BRESEQ :: Evidence


| New junction evidence | | | | | | | | | | | |
| --- | --- | --- | --- | --- | --- | --- | --- | --- | --- | --- | --- |
|  | | seq id | position | reads (cov) | reads (cov) | score | skew | freq | annotation | gene | product |
| \* | ? | NC\_001604.1 | = 7972 | 2034 (1.180) | 21 (0.010) | 20/588 | 34.7 | 1.1% | intergenic (–/–) | *–/–* | –/– |
|  | ? | NC\_001604.1 | 8524 = | 1958 (1.160) |  |  |  |  | intergenic (–/–) | *–/–* | –/– |
| Rejected: Coverage evenness skew score above cutoff. | | | | | | | | | | | |
| Rejected: Frequency below/above cutoff threshold. | | | | | | | | | | | |

Only 100 of 1973 total aligned reads displayed.  

`GGTGTATAGCTTGCTTAGAGAAAGATGACCGTTATCCACACACCTGTAACAAAGGAGCTAACGATATGACCGAACGTGAACAAGAGATGATCATTAAGTTGATAGACAATAATGAAGGTCGCCCAGATGATTTGAATGGCTGCGGTATTCTCTGCTCCAATGTCCCTTGCCACCTCTGCCCCGCAAATAACGATCAAAAGATAACCTTAGGTGAAATCCGAGCGATGGACCCACGTAAACCACATCTGAATAAACCTGAGGTAACTCC‑TACAGATGACCAGCCTTCCGCTGAGACAATCGAAGGTGTCACTAAGCCTTCCCACTACATGCTGTTTGACGACATTGAGGCTATCGAAGTGATTGCTCGTTCAATGACCGTTGAGCAGTTCAAGGGATACTGCTTCGGTAACATCTTAAAGTACAGACTACGTGCTGGTAAGAAGTCAGAGTTAGCGTACTTAGAGAAAGACCTAGCGAAAGCAGACTTCTATAAAGAACTCTTTGAGAAACATAAGGATAAATGTTATGCATAACTTCAAGTCAACCCCACCTGCCGACAGCCTATCTGATGACTTCACATCTTGCTCAGAGTGGTGCCGAA  >  NC_001604.1/8224‑8824`  

                                                                                                                                                                                                                                                                                                             `|`                                                                                                                                                                                                                                                                                                              
       `aGCTTGCTTAGAGAAAGATGACCGTTATCCACACACCTGTAACAAAGGAGCTAACGATATGACCGAACGTGAACAAGAGATGATCATTAAGTTGCTAGACAATAATGAAGGTCGCCCAGATGATTTGAATGGCTGCGGTATTCTCTGCTCCAATGTCCCTTGCCACCTCTGCCCCGCAACTAACGTTCAAAAGATAACCTTAGGTGAAATCCGAGCGATGGACCCACGTAAACCACATCTGAATAAACCTGAGGTAACTCC‑TACAGATAACCAGCCTTCCGCTGAGACAATCGAAGgtgta                                                                                                                                                                                                                                                                                                       >  1:240643/1‑300 (MQ=255)`  
              `ggggctcggaaatgtgtataagagacagccTGTAACAAAGGAGCTAACGATATGACAGAACGTGAAAAAGAGATGATCATTAAGTTGATAGACAATAATGAAGGTCGCCCAGATGATTTGAATGGCTGCGGTATTCTCTGCTCCAATGTCCCTTCCCAACTCTGCCCCGCAAATAACGATCAAAAGATAACCTTAGGTGAAATCCGAGCGATGGACCCACGT‑AACCACATCTGAATAAACCTGAGGTAACTCC‑TACAGATGACCAGCCTTCCGCTGAGACAATCGAAGGTGTCACTAAGcc                                                                                                                                                                                                                                                                                               <  1:26737/273‑1 (MQ=255)`  
               `tAGAGAAAGATGACCGTTATCCACACACCTGTAACAAAGGAGCTAACGATATGACCGAACGTGAACAAGAGATGATCATTAAGTTGATAGACAATAATGAAGGTCGCCCAGATGATTTGAATGGCTGCGGTATTCTCTGCTCCAATGTCCCTTGCCACCTCTGCCCCGCAAATAACGATCAAAAGATAACCTTAGGTGAAATCCGAGCGATGGACCCACGTAAACCACATCTGAATAAACCTGAGGTAACTCC‑TACAGATGACCAGCCTTCCGCTGAGACAATCGAAGGTGTCACTAAGcc                                                                                                                                                                                                                                                                                               <  1:50100/301‑1 (MQ=255)`  
                    `gagacagACCGTTAACCACACACCTGTAACAAAGGAGCTAACGATATGACCCAACGTGAACAAGAGATGAGCATTAAGTTGCTAGAAAATAATGAAGGTCTCCCAGATGATTTGAATGGCTGCGGTATTTTCTCCTCCAATGTCCCTTGCCACCTCTGCCCCGTAAATAACGATCAAAAGATAACCTTAGGTGAAATCCGAGCGATGGACCCACGTAAACCACATCTGAATAAACCTGAGGTAACTCC‑TACAGATGACCAGCCTTCCGCTGAGACAATCGAAGGTGTCACTAAGCCTTccc                                                                                                                                                                                                                                                                                          <  1:144886/295‑1 (MQ=255)`  
                    `aaaGATGACCGTTATCCACACACCTGTAACAAAGGAGCTAACGATATGACCGAACGTGAACAAGAGATGATCATTAAGTTGATAGACAATAATGAAGGTCGCCCAGATGATTTGAATGGCTGCGGTATTCTCTGCTCCAATGTCCCTTGCCACCTCTGCCCCGCAAATAACGATCAAAAGATAACCTTAGGTGAAATCCGAGCGATGGACCCACGTAAACCACATCTGAATAAACCTGAGGTAACTCC‑TACAGATGACCAGCCTTCCGCTGAGACAATCGAAGGTGTCACTAAGCCTTccc                                                                                                                                                                                                                                                                                          <  1:273450/301‑1 (MQ=255)`  
                         `tGACCGTTATCCAACAACCTGTAGCAAAGGAGCTAACGATATGACCGAACGTGAACAAGAGATGATCATTAAGTTGATAGACAATAATGAAGGTCGCCCAGATGATTTGAATGGCTGCGGTATTCTCTGCTCCAATGTCCCTTGCCACCTCTGCCCCGCAAATAACGATCAAAAGATAACCTTAGGTGAAATCCGAGCGATGGACCCACGTAAACCACATCTGAATAAACCTGAGGTAACTCC‑TACAGATGACCAGCCTTCCGCTGAGACAATCGAAGGTGTCACTAAGCCTTCCCACTAc                                                                                                                                                                                                                                                                                     <  1:219371/301‑1 (MQ=255)`  
                             `cGTTATCCACACACCTGTAACAAAGGAGCTAACGATATGACCGAACGTGAACAAGAGATGATCATTAAGTTGATAGACAATAATGAAGGTCGCCCAGATGATTTGAATGGCTGCGGTATTCTCTGCTCCAATGTCCCTTGCCACCTCTGCCCCGCAAATAACGATCAAAAGATAACCTTAGGTGAAATCCGAGCGATGGACCCACGTAAACCACATCTGAATAAACCTGAGGTAACTCC‑TACAGATGACCAGCCTTCCGCTGAGACAATCGAAGGTGTCACTAAGCCTTCCCACTACATGc                                                                                                                                                                                                                                                                                 <  1:259376/301‑1 (MQ=255)`  
                             `cGTTATCCACACACCTGTAACAAAGGAGCTAACGATATGACCGAACGTGAACAAGAGATGATCATTAAGTTGATAGACAATAATGAAGGTCGCCCAGATGATTTGAATGGCTGCGGTATTCTCTGCTCCAATGTCCCTTGCCACCTCTGCCCCGCAAATAACGATCAAAAGATAACCTTAGGTGAAATCCGAGCGATGGACCCACGTAAACCACATCTGAATAAACCTGAGGTAACTCC‑TACAGATGACCAGCCTTCCGCTGAGACAATCGAAGGTGTCACTAAGCCTTCCCACTACATGc                                                                                                                                                                                                                                                                                 <  1:105776/301‑1 (MQ=255)`  
                               `ttATCCACACACCTGTAACAAAGGAGCTAACGATATGACCGAACGTGAACAAGAGATGATCATTAAGTTGATAGACAATAATGAAGGTCGCCCAGATGATTTGAATGGCTGCGGTATTCTCTGCTCCAATGTCCCTTGCCACCTCTGCCCCGCAAATAACGATCAAAAGATAACCTTAGGTGAAATCCGAGCGATGGACCCACGTAAACCACATCTGAATAAACCTGAGGTAACTCC‑TACAGATGACCAGCCTTCCGCTGAGACAATCGAAGGTGTCACTAAGCCTTCCCACTACAtctgt                                                                                                                                                                                                                                                                               >  1:123836/1‑297 (MQ=255)`  
                               `ttATCCACACACCTGTAACAAAGGAGCTAACGATATGACAGAACGTGAACAAGAGATGATCATTAAGTTGATAGACAATAATGAAGGTCGCCCAGATGATTTGAATGGCTGCGGTATTCTCTGCTCCAATGTCCCTTGCCACCTCTGCCCCGCAAATAACGATCAAAAGATAACCTTAGGTGAAATCCGAGCGATGGACCCACGTAAACCACATCTGAATAAACCTGAGGTAACTCC‑TACAGATGACCAGCCTTCCGCTGAGACAATCGAAGGTGTCACTAAGCCTTCCCACTACATGCTg                                                                                                                                                                                                                                                                               >  1:150234/1‑301 (MQ=255)`  
                                 `aTCCACACACCTGTAACAAAGGAGCTAACGATATGACCGAACGTGAACAATAGATGATCATTAAGTTGATAGACAATAATGAAGGTCGCCCAGATGATTTGAATGGCTGCGGTATTCTCTGCTCCAATGTCCCTTGCCACCTCTGCCCCGCAAATAACGATCAAAAGATAACCTTAGGTGAAATCCGAGCGATGGACCCACGTAAACCACATCTGAATAAACCTGAGGTAACTCC‑TACAGATGACCAGCCTTCCGCTGAGACAATCGAAGGTGTCACTAAGCCTTCCCACTACATGCTGtt                                                                                                                                                                                                                                                                             >  1:70405/1‑301 (MQ=255)`  
                                     `cagcacCTGTAACAAAGGAGCTAACGATATGACCGAACGTGAACAAGAGATGATCATTAAGTTGATAGACAATAATGAAGGTCGCCCAGATGATTTGAATGGCTGCGGTATTCTCTGCTCCAATGTCCCTTGCCACCTCTGCCCCGAAAATAACGATCAAAAGATAACCTTAGGTGAAATCCGAGCGATGGACCCACGTAAACCACATCTGAATAAACCTGAGGTAACTCC‑TACAGATGACCAGCCTTCCGCTGAGACAATCGAAGGTGTCACTAAGCCTTCCCACTACATGCTGTTTgac                                                                                                                                                                                                                                                                         <  1:257391/298‑1 (MQ=255)`  
                                      `cggagatgtgtataagagacagaCGATATGACCGAACGTGAACAAGAGATGATCATTAAGTTGATAGACAATAATGAAGGTCGCCCAGATGATTTGAATGGCTGCGGTATTCTCTGCTCCAATGTCCCTTGCCACCTCTGCCCCGCAAATAACGATCAAAAGATAACCTTAGGTGAAATCCGAGCGATGGACCCACGTAAACCACATCTGAATAAACCTGAGGTAACTCC‑TACAGATGACCAGCCTTCCGCTGAGACAATCGAAGGTGTCACTAAGCCTTCCCACTACATGCTGTTTgacg                                                                                                                                                                                                                                                                        <  1:317702/279‑1 (MQ=255)`  
                                       `cagccTGGAACAAAGGAGCTAACGATATGACCGAACGTGAACAAGGGAGGATCATTAGGTTGATAGACAATAATGAAGGTCGCCCAGATGATTTGAATGGCTGCGGTATTCTCTGCTCCAATGGCCCTTGCCACCTCTGCCCCGCAAATAACGATCAAAAGATAACCTTAGGTGAAATCCGAGCGATGGACCCACGTAAACCACATCTGAATAAAACTGAGGTAACGCC‑TACAGATGACCAGCCTTCCGCTGAGACAATCGAAGGTGTCACTAAGCCTTCCCACTACATGCTGTTTgacga                                                                                                                                                                                                                                                                       <  1:160006/298‑1 (MQ=255)`  
                                       `aaacCTGTAACAAAGGAGCTAACGATATGACCGAACGTGAACAAGAGATGATCATTAAGTTGATAGACAATAATGAAGGTCGCCCAGATGATTTGAATGGCTGCGGTATTCTCTGCTCCAATGTCCCTTGCCACCTCTGCCCCGCAAATAACGATCAAAAGATAACCTTAGGTGAAATCCGAGCGATGGACCCACGTAAACCACATCTGAATAAACCTGAGGTAACTCC‑TACAGATGACCAGCCTTCCGCTGAGACAATCGAAGGTGTCACTAAGCCTTCCCACTACATGCTGTTTgacga                                                                                                                                                                                                                                                                       <  1:169072/299‑1 (MQ=255)`  
                                        `aacCTGTAACAAAGGAGCTAACGATATGACCGAACGTGAACAAGAGATGATCATTAAGTTGATAGACAATAATGAAGGTCGCCCAGATTATCTGAATGGCTGCGGTATTCTCTGCTCCAATGTCCCTTGCCACCTCTGCCCCGCAAATAACGATCAAAAGATAACCTTAGGTGAAATCCGAGCGATGGACCCACGTAAACCACATCTGAATAAACCTGAGGTAACTCC‑TACAGATGACCAGCCTTCCGATGAGACAATCGAAGGTGTCACTAAGCCTTCCCACTACATGCTGTTTgacgac                                                                                                                                                                                                                                                                      <  1:73411/300‑1 (MQ=255)`  
                                          `ccTGTAACAAAGGAGCTAACGATATGACCGAACGTGAACAAGAGATGATCATTAAGTTGATAGACAATAATGAAGGTCGCCCAGATGATTTGAATGGCTGCGGTATTCTCTGCTCCAATGTCCCTTGCCACCTCTGCCCCGCAAAAAACGATCAAAAGATAACCTTAGGTGAAATCCGAGCGATGGACCCACGTAAACCACATCTGAATAAACCTGAGGTAACTCC‑TACAGATGACCAGCCTTCCGCTGAGACAATCGAAGGTGTCACTAAGCCTTCCCACTACATGCTGTTTgacgacct                                                                                                                                                                                                                                                                    >  1:225260/1‑299 (MQ=255)`  
                                             `gTAACAAATGAGCTAACGATATGACCGAACGTGAACAAGAGATGATCATTAAGTTGATAGACAATAATGAAGGTCGCCCAGATGATTTGAATGGCTGCGGTATTCTCTGCTCCAATGTCCCTTGCCACCTCTGCCCCGCAAATAACGATCAAAAGATAACCTTAGGTGAAATCCGAGCGATGGACCCACGTAAACCACATCTGAATAAACCTGAGGTAACTCC‑TACAGATGACCAGCCTTCCGCTGAGACAATCGAAGGTGTCACTAAGCCTTCCCACTACATGCTGTTTGACGACATTGa                                                                                                                                                                                                                                                                 >  1:108778/1‑301 (MQ=255)`  
                                               `aaCAAAGGAGCTAACGATATGACCGAACGTGAACAAGAGATGATCATTAAGTTGATAGACAATAATGAAGGTCGCCCAGATGATTTGAATGGCTGCGGTATTCTCTGCTCCAATGTCCCTTGCCACCTCTGCCCCGCAAATAACGATCAAAAGATAACCTTAGGTGAAATCCGAGCGATGGACCCACGTAAACCACATCTGAATAAACCTGAGGTAACTCC‑TACAGATGACCAGCCTTCCGCTGAGACAATCGAAGGTGTCACTAAGCCTTCCCACTACATGCTGTTTGACGACATTGAgg                                                                                                                                                                                                                                                               <  1:76645/301‑1 (MQ=255)`  
                                               `aaCAAAGGAGCTAACGATATGACCGAACGTGAACAAGAGATGATCATTAAGTTGATAGACAATAATGAAGGTCGCCCAGATGATTTGAATGGCTGCGGTATTCTCTGCTCCAATGTCCCTTGCCACCTCTGCCCCGCAAATAACGATCAAAAGATAACCTTAGGTGAAATCCGAGCGATGGACCCACGTAAACCACATCTGAATAAACCTGAGGTAACTCC‑TACAGATGACCAGCCTTCCGCTGAGACAATCGAAGGTGTCACTAAGCCTTCCCACTACACGCTGTTTGACGACATTGAgg                                                                                                                                                                                                                                                               <  1:53960/301‑1 (MQ=255)`  
                                                        `gacagcGATATGACCGAACGTGAACAAGAGATGATCATTAAGTTGATAGACAATAATGAAGGTCGCCCAGATGATTTGAATGGCTGCGGTATTCTCTGCTCCAATGTCCCTTGCCACCTCTGCCCCGCAAATAACGATCAAAAGATAACCTTAGGTGAAATCCGAGCGATGGACCCACGTAAACCACATCTGAATAAACCTGAGGTAACTCC‑TACAGATGACCAGCCTTCCGCTGAGACAATCGAAGGTGTCACTAAGCCTTCCCACTACATGCTGTTTGACGACATTGAGGCTATCGAAg                                                                                                                                                                                                                                                      <  1:6779/296‑1 (MQ=255)`  
                                                                        `aaCGTGAACAAGAGATGATCATTAAGTTGATAGACAATAATGAAGGTCGCCCAGATGATTTGAATGGCTGCGGTATTCTCTGCTCCAATGTCCCTTGCCACCTCTGCCCCGCAAATAACGATCAAAAGATAACCTTAGGTGAAATCCGAGCGATGGACCCACGTAAACCACATCTGAATAAACCTGAGGTAACTCC‑TACAGATGACCAGCCTTCCGCTGAGACAATCGAAGGTGTCACTAAGCCTTCCCACTACATGCTGTTTGACGACATTGAGGcctgtctcttatacacatctccgag                                                                                                                                                                                                                                      >  1:138565/1‑277 (MQ=255)`  
                                                                        `aaCGTGAACAAGAGATGATAATTAAGTTGATAGACAATAATGAAGGTCGCCCAGATGATTTGAATGGCTGCTGTATTCTTTGCTCCAATGTACCTTGCCACCTCTGCCCCGCAAATAACGATCAAAAGATAACCTTAGGTGAAATCCGAGCGATGGACCTACGTTCACCACATCTGAATAAACCTGAGGTAACTCC‑TACAGATGACAAGCCTTCGGCTTCACTCATCCAAGGTGTCACTCAGACGTCCCATTAAATGCTGTTTGACGACATTAAGTCTATCGAAGTTATTGCTCATTAAAt                                                                                                                                                                                                                                      >  1:347355/1‑301 (MQ=255)`  
                                                                           `gTGAACAAGAGATGATCATTAAGTTGATATACAATAATGAAGGTCGCCCAGATGATTTGAATGGCTGCGGTATTCTCTGCTCCAATGTCCCTTGCCACCTCTGCCCCGCAAATAACGATCAAAAGATAACCTTAGGTGAAATCCGAGCGATGGACCCACGTAAACCACATCTGAATAAACCTGAGGTAACTCC‑TACAGATGACCAGCCTTCCGCTGAGACAATCGAAGGTGTCACTAAGCCTTCCCACTACATGCTGTTTGACGACATTGAGGCTATCGAAGTGATTGCTCGTTCAATGAc                                                                                                                                                                                                                                   <  1:93116/301‑1 (MQ=255)`  
                                                                           `gTGAACAAGAGATGATCATTAAGTTGATAGACAATAATGAAGGTCGCCCAGATGATTTGAATGGCTGCGGTATTCTCTGCTCCAATGTCCCTTGCCACCTCTGCCCCGCAAATAACGATCAAAAGATAACCTTAGGTGAAATCCGAGCGATGGACCCACGTAAACCACATCTGAATAAACCTGAGGTAACTCC‑TACAGATGACCAGCCTTCCGCTGAGACAATCGAAGGTGTCACTAAGCCTTCCCACTACATGCTGTTTGACGACATTGAGGCTATCGAAGTGATTGCTCGTTCAATGAc                                                                                                                                                                                                                                   >  1:250057/1‑301 (MQ=255)`  
                                                                             `gAACAAGAGATGATCATTAAGTTGATAGACAATAATGAAGGTCGCCCAGATGATTTGAATGGCTGCGGTATTCTCTGCTCCAATGTCCCTTGCCACCTCTGCCCCGCAAATAACGATCAAAAGATAACCTTAGGTGAAATCCGAGCGATGGACCCACGTAAACCACATCTGAATAAACCTGAGGTAACTCC‑TACAGATGACCAGCCTTCCGCTGAGACAATCGAAGGTGTCACTAAGCCTTCCCACTACATGCTGTTTGACGACATTGAGGCTATCGAAGTGATTGCTCGTTCAATGACCg                                                                                                                                                                                                                                 >  1:315814/1‑301 (MQ=255)`  
                                                                              `aaCAAGAGATGATCATTAAGTTGATAGACAATAATGAAGGTCGCCCAGATGATTTGAATGGCTGCGGTATTCTCTGCTCCAATGTCCCTTGCCACCTCTGCCCCGCAAATAACGATCAAAAGATAACCTTAGGTGAAATCCGAGCGATGGACCCACGTAAACCACATCTGAATAAACCTGAGGTAACTCC‑TACAGATGACCAGCCTTCCGCTGAGACAATCGAAGGTGTCACTAAGCCTTCCCACTACATGCTGTTTGACGACATTGAGGCTATCGAAGTGATTGCTCGTTCAATGACCGt                                                                                                                                                                                                                                >  1:171401/1‑301 (MQ=255)`  
                                                                                `cAAGAGATGATCATTAAGTTGATAGACAATAATGAAGGTCGCCCAGATGATTTGAATGGCTGCGGTATTCTCTGCTCCAATGTCCCTTGCCACCTCTGCCCCGCAAATAACGATCAAAAGATAACCTTAGGTGAAATCCGAGCGATGGACCCACGTAAACCACATCTGAATAAACCTGAGGTAACTCC‑TACAGATGACCAGCCTTCCGCTGAGACAATCGAAGGTGTCACTAAGCCTTCCCACTACATGCTGTTTGACGACATTGAGGCTATCGAAGTGATTGCTCGTTCAATGACCGttt                                                                                                                                                                                                                              >  1:196633/1‑300 (MQ=255)`  
                                                                                           `cATTAAGTTGATAGACAATAATGAAGGTCGCCCAGATGATTTGAATGGCTGCGGTATTCTCTGCTCCAATGTCCCTTGCCACCTCTGCCCCGCAAATAACGATCAAAAGATAACCTTAGGTGAAATCCGAGCGATGGACCCACGTAAACCACATCTGAATAAACCTGAGGTAACTCC‑TACAGATGACCAGCCTTCCGCTGAGACAATCGAAGGTGTCACTAAGCCTTCCCACTACATGCTGTTTGACGACATTGAGGCTATCGAAGTGATTGCTCGTTCAATGACCGTTGAGCAGTTCAAg                                                                                                                                                                                                                   >  1:156862/1‑301 (MQ=255)`  
                                                                                             `ttAAGTTGATAGACAATAATGAAGGTCGCCCAGATGATTTGAATGGCTGCGGTATTCTCTGCTCCAATGTCCCTTGCCACCTCTGCCCCGCAAATAACGATCAAAAGATAACCTTAGGTGAGATCCGAGCGATGGACCCACGTAAACCACATCTGAATAAACCTGAGGTAACTCC‑TACAGATGACCAGCCTTCCGCTGAGACAATCGAAGGTGTCACTAAGCCTTCCCACTACATGCTGTTTGACGACATTGAGGCTATCGAAGTGATTGCTCGTTCAATGACCGTTGAGCAGTTCAAggg                                                                                                                                                                                                                 <  1:42960/301‑1 (MQ=255)`  
                                                                                             `ttAAGTTGATAGACAATAATGAAGGTCGCCCAGATGATTTGAATGGCTGCGGTATTCTCTGCTCCAATGTCCCTTGCCACCTCTGCCCCGCAAATAACGATCAAAAGATAACCTTAGGTGAAATCCGAGCGATGGACCCACGTAAACCACATCTGAATAAACCTGAGGTAACTCC‑TACAGATGACCAGCCTTCCGCTGAGACAATCGAAGGTGTCACTAAGCCTTCCCACTACATGCTGTTTGACGACATTGAGGCTATCGAAGTGATTGCTCGTTCAATGACCGTTGAGCAGTTCAAggg                                                                                                                                                                                                                 <  1:237667/301‑1 (MQ=255)`  
                                                                                             `ttAAGTTGATAGACAATAATGAAGGTCGCCCAGATGATTTGAATGGCTGCGGTATTCTCTGCTCCAATGTCCCTTGCCACCTCTGCCCCGCAAATAACGATCAAAAGATAACCTTAGGTGAAATCCGAGCGATGGACCCACGTAAACCACATCTGAATAAACCTGAGGTAACTCC‑TACAGATGACCAGCCTTCCGCTGAGACAATCGAAGGTGTCACTAAGCCTTCCCACTACATGCTGTTTGACGACATTGAGGCTATCGAAGTGATTGCTCGTTCAATGACCGTTGAGCAGTTCAAggg                                                                                                                                                                                                                 <  1:231069/301‑1 (MQ=255)`  
                                                                                             `ttAAGTTGATAGACAATAATGAAGGTCGCCCAGATGATTTGAATGGCTGCGGTATTCTCTGCTCCAATGTCCCTTGCCACCTCTGCCCCGCAAATAACGATCAAAAGATAACCTTAGGTGAAATCCGAGCGATGGACCCACGTAAACCACATCTGAATAAACCTGAGGTAACTCC‑TACAGATGACCAGCCTTCCGATGAGACAATCGAAGGTGTCACTAAGCCTTCCCACTACATGCTGTTTGACGACATTGAGGCTATCGAAGTGATTGCTCGTTCAATGACCGTTGAGCAGTTCAAggg                                                                                                                                                                                                                 <  1:17749/301‑1 (MQ=255)`  
                                                                                             `ttAAGTTGATAGACAATAATGAAGGTCGCCCAGATGATTTGAATGGCTGCGGTATTCTCTGCTCCAATGTCCATTGCCACCTCTGCCCCGCAAATAACGATCAAAAGATAACCTTAGGTGAAATCCGAGCGATGGACCCACGTAAACCACATCTGAATAAACCTGAGGTAACTCC‑TACAGATGACCAGCCTTCCGCTGAGACAATCGAAGGTGTCACTAAGCCTTCCCACTACATGCTGTTTGACGACATTGAGGCTATCGAAGTGATTGCTCGTTCAATGACCGTTGAGCAGTTCAAggg                                                                                                                                                                                                                 <  1:132762/301‑1 (MQ=255)`  
                                                                                             `ttAAGTTGATAGAAAATAATGAAGGTCGCCCAGATGATTTGAATGGCTGCGGTATTCTCTGCTCCAATGTCCCTTGCCACCTCTGCCCCGCAAATAACGATCAAAAGATAACCTTAGGTGAAATCCGAGCGATGGACCCACGTAAACCACATCTGAATAAACCTGAGGTAACTCC‑TACAGATGACCAGCCTTCCGCTGAGACAATCGAAGGTGTCACTAAGCCTTCCCACTACATGCTGTTTGACGACATTGAGGCTATCGAAGTGATTGCTCGTTCAATGACCGTTGAGCAGTTCAAggg                                                                                                                                                                                                                 <  1:3109/301‑1 (MQ=255)`  
                                                                                                 `gTTGATAGACAATAATGAAGGTCGCCCAGATGATTTGAATGGCTGCGGTATTCTCTGCTCCAATGTCCCTTGCCACCTCTGCCCCGCAAATAACGATCAAAAGATAACCTTAGGTGAAATCCGAGCGATGGACCCACGTACACCACATCTGAATAAACCTGAGGTAACTCC‑TACAGATGACCAGCCTTCCGCTGAGACAATCGAAGGTGTCACTAAGCCTTCCCACTACATGCTGTTTGACGACATTGAGGCTATCGAAGTGATTGCTCGTTCAATGACCGTTGAGCAGTTCAAGGGATAc                                                                                                                                                                                                             <  1:39738/301‑1 (MQ=255)`  
                                                                                                          `cAATAATGAAGGTCGCCCAGATGATTTGAATGGCTGCGGTATTCTCTGCTCCAATGTCCCTTGCCACCTCTGCCCCGCAAATAACGATCAAAAGATAACCTTAGGTGAAATCCGAGCGATGGATCCACGTAAACCACATCTGAATAAACCTGAGGTAACTCC‑TACAGATGACCAGCCTTCCGCTGAGACAATCGAAGGTGTCACTAAGCCTTCCCACTACATGCTGTTTCACGACATTGAGGCTATCGAAGTGATTGCTCGTTCAATGACCGTTGAGCAGTTCAAGGGATACTGCTTCGGt                                                                                                                                                                                                    >  1:287908/1‑301 (MQ=255)`  
                                                                                                          `cAATAATGAAGGTCGCCCAGATGATTTGAATGGCTGCGGTATTCTCTGCTCCAATGTCCCTTGCCACCTCTGCCCCGCAAATAACGATCAAAAGATAACCTTAGGTGAAATCCGAGCGATGGACCCACGTAAACCACATCTGAATAAACCTGAGGTAACTCC‑TACAGATGACCAGCCTTCCGCTGAGACAATCGAAGGTGTCACTAAGCCTTCCCACTACATGCTGTTGGACGACATTGAGGCTATCGAAGTGATTGCTCGTTCAATGACCGTTGAGCAGTTCAAGGGATACTGCTTCggg                                                                                                                                                                                                    >  1:9565/1‑300 (MQ=255)`  
                                                                                                           `aataatGAATGTCGCCCAGATGATTTGAATGGCTGCGGTATTCTCTGCTCCAATGTCCCTTGCCACCTCTGCCCCGCAAATAACGATCAAAAGATAACCTTAGGTGAAATCCGAGCGATGGACCCACGTAAACCACATCTGAATAAACCTGAGGTAACTCC‑TACAGATGACCAGCCTTCCGCTGAGACAATCGAAGGTGTCACTAAGCCTTCCCACTACATGCTGTTTGACGACATTGAGGCTATCGAAGTGATTGCTCGTTCAATGACCGTTGAGCAGTTCAAGGGATACTGCTTCGGTa                                                                                                                                                                                                   <  1:335185/301‑1 (MQ=255)`  
                                                                                                           `aataatGAAGGTCGCCCAGATGATTTGAATGGCTGCGGTATTCTCTGCTCCAATGTCCCTTGCCACCTCTGCCCCGCAAATAACGATCAAAAGATAACCTTAGGTGAAATCCGAGCGATGGACCCACGTAAACCACATCTGAATAAACCTGAGGTAACTCC‑TACAGATGACCAGCCTTCCGCTGAGACAATCGAAGGTGTCACTAAGCCTTCCCACTACATGCTGTTTGACGACATTGAGGCTATCGAAGTGATTGCTCGTTCAATGACCGTTGAGCAGTTCAAGGGATACTGCTTCGGTa                                                                                                                                                                                                   <  1:129179/301‑1 (MQ=255)`  
                                                                                                           `aataagGAAGGTCGCCGAGATGATTTGAATGGCTGCGGTATTCTCTGCTCCAATGTCCCTTGCCACCTCTGCCCCGCAAATAACGATCAAAAGATAACCTTAGGTGAAATCCGAGCGATGGACCCACGTAAACCACATCTGAATAAACCTGAGGTAACTCC‑TACAGATGACCAGCCTTCCGCTGAGACAATCGAAGGTGTCACTAAGCCTTCCCACTACATGCTGTTTGACGACATTGAGGCTATCGAAGTGATTGCTCGTTCAATGACCGTTGAGCAGTTCAAGGGATACTGCTTCGGTa                                                                                                                                                                                                   <  1:216180/301‑1 (MQ=255)`  
                                                                                                            `ataatGAAGGTCGCCCAGATGATTTGAATGGCTGCGGTATTCTCTGCTCCAATGTCCCTTGCCACCTCTGCCCCGCAAATAACGATCAAAAGATAACCTTAGGTGAAATCCGAGCGATGGACCCACGTAAACCACATCTGAATAAACCTGAGGTAACTCC‑TACAGATGACCAGCCTTCCGCCGAGACAATCGAAGGTGTCACTAAGCCTTCCCACTACATGCTGTTTGACGACATTGAGGCTATCGAAGTGATTGCTCGTTCAATGACCGTTGAGCAGTTCAAGGGATACTGCTTCGGTaa                                                                                                                                                                                                  >  1:87736/1‑301 (MQ=255)`  
                                                                                                            `ataatGAAGGTCGCCCAGATGATTTGAATGGCTGCGGAATTCTCTGCTCCAATGTCCCTTGCCACCTCTGCCCCGCAAATAACGATCAAAAGATAACCTTAGGTGAAATCCGAGCGATGGACCCACGTAAACCACATCTGAATAAACCTGAGGTAACTCC‑TACAGATGACCAGCCTTCCGCTGAGACAATCGAAGGTGTCACTAAGCCTTCCCACTACATGCTGTTTGACGACATTGAGGCTATCGAAGTGATTGCTCGTTCAATGACCGTTGAGCAGTTCAAGGGATACTGCTTCGGTaa                                                                                                                                                                                                  >  1:111948/1‑301 (MQ=255)`  
                                                                                                            `ataatGAAGGTCGCCCAGATGATTTGAATGGCTGAGGTATTCTCTGCTCCAATGTCCCTTGCCACCTCTGCCCCGCAAATAACGATCAAAAGATAACCTTAGGTGAAATCCGAGCGATGGACCCACGTAAACCACATCTGAATAAACCTGAGGTAACTCC‑TACAGATGACCAGCCTTCCGCTGAGACAATCGAAGGTGTCACTAAGCCTTCCCACTACTTGCTGTTTGCCAACATTAAGGCTATCGAAGTGATTGCTCGTTCAATGACCGTTGAGAAGTTCAAGGGATCCTGCTTCGGTac                                                                                                                                                                                                  >  1:173793/1‑300 (MQ=255)`  
                                                                                                              `aatGAAGGTCGCCCAGATGATTTGAATGGTTGCGGTATTCCCTGCTCCAATGTCCCTTGCCACCTCTGCCCCGCAAATAACGATCAAAAGAAAACCTTAGGTGAAATCCGAGCGATGGACCCACGTAAACCACATCTGAATAAACCTGAGGTAACTAC‑AACAGATGACCAGACTACCGCTGAGACAATAGAAGGAGTCACTAAGCCTTCCCACTACATGCTGTTTGAAGACATTGAGGCTATCGAAGTGATTGATCGTTCAATGACCGTTGAGCAGTTCATgggctgtgtcatatacacat                                                                                                                                                                                                >  1:12045/1‑284 (MQ=255)`  
                                                                                                                   `aGGTCGCCCAGATGATTTGAATGGCTGCGGTATTCTCTGCTCCAATGTCCCTTGCCACCTCTGCCCCGCAAATAACGATCAAAAGATAACCTTAGGTGAAATCCGAGCGATGGACCCACGTAAACCACATCTGAATAAACCTGAGGTAACTCC‑TACAGATGACCAGCCTTCCGCTGAGACAATCGAAGGTGTCACTAAGCCTTCCCACTACATGCTGTTTGACGACATTGAGGCTATCGAAGTGATTGCTCGTTCAATGACCGTTGAGCAGTTCAAgggctgtctcttatacacatctccg                                                                                                                                                                                           >  1:283422/1‑279 (MQ=255)`  
                                                                                                                      `cagCCCAGATGATTTGAATGGCTGCGGTATTCTCTGCTCCAATGTCCCTTGCCACCTCTGCCCCGCAAATAACGATCAAAAGATAACCTTAGGTGAAATCCGAGCGATGGACCCACGTAAACCACATCTGAATAAACCTGAGGTAACTCC‑TACAGATGACCAGCCTTCCGCTGAGACAATCGAAGGTGTCACTAAGCCTTCCCACTACATGCTGTTTGACGACATTGAGGCTATCGAAGTGATTGCTCGTTCAATGACCGTTGAGCAGTTCAAGGGATACTGCTTCGGTAACATCTTAAAg                                                                                                                                                                                        <  1:280680/299‑1 (MQ=255)`  
                                                                                                                         `gggagatgtgtataagagaccgggTATTCTCTGCTCCAATGTCACTTGCCACCTCTGCCCCGCAAATAACGATCAAAAGATAACCTTAGGTGAAATCCGAGCGATGGACCCACGTAAACCACATCTGAATAAACCTGAGGTAACTCC‑TACAGATGACCAGCCTTCCGCTGAGACAATCGAAGGAGTCACTAAGCCTTCCCACTACATGCTGTTTGACGACATTGAGGCTATCGAAGTGATTGCTCGTTCAATGACCGTTGAGCAGTTCAAGGGATACTGCTTCGGTAACATCTTAAAGTAc                                                                                                                                                                                     <  1:320591/279‑1 (MQ=255)`  
                                                                                                                         `cccAGATGATTTGAATGGCTGCGGTATTCTCTGCTCCAATGTCCCTTGCCACCTCTGCCCCGCAAATAACGATCAAAAGATAACCTTAGGTGAAATCCGAGCGATGGACCCACGTAAACCACATCTGAATAAACCTGAGGTAACTCC‑TACAGATGACCAGCCTTCCGCTGAGACAATCGAAGGTGTCACTAAGCCTTCTCACTACATGCTGTTTGACGACATTGAGGCTATCGAAGTGATTGCTCGTTCAATGACCGTTGAGCAGTTCAAGGGATACTGCTTCGGTAACATCTTAAAGTAc                                                                                                                                                                                     <  1:190342/301‑1 (MQ=255)`  
                                                                                                                              `atgatTTGAAAGGCTGCGGTATTCTCTGCTCCAATGTCCCTTGCCACCTCTGCCCCGCAAATAACGATCAAAAGATAACCTTAGGTGAAATCCGAGCGATGGACCCACGTAAACCACATCTGAATAAACCTGAGGTAACTCC‑TACAGATGACCAGCCTTCCGCTGAGACAATCGAAGGTGTCACTAAGCCTTCCCACTACATGCTGTTTGACGACATTGAGGCTATCGAAGTGATTGCTCGTTCAATGACCGTTGAGCAGTTCAAGGGATACTGCTTCGGTAACATCTTAAAGTACAGACt                                                                                                                                                                                <  1:212307/301‑1 (MQ=255)`  
                                                                                                                               `tgatTTGAATGGCTGCGGTATTCTCTGCTCCAATGTCCCTTGCCACCTCTGCCCCGCAAATAACGATCAAAAGATAACCTTAGGTGAAATCCGAGCGATGGACCCACGTAAACCACATCTGAATAAACCTGAGGTAACTCC‑TACAGATGACCAGCCTTCCGCTGAGACAATCGAAGGTGTCACTAAGCCTTCCCACTACATGCTGTTTGACGACATTGAGGCTATCGAAGTGATTGCTCGTTCAATGACCGTTGAGCAGTTCAAGGGATAcctgtctcttatacacatctccgagcccacg                                                                                                                                                                               >  1:291258/1‑271 (MQ=255)`  
                                                                                                                                `gatTTGAATGGCTGCGATATTCTCTGCTCCAATGTCCCTTGCCACCTCTGCCCCGCAAATAACGATCAAAAGATAACCTTAGGTGAAATCCGAGCGATGGACCCACGTAAACCACATCTGAATAAACCTGAGGTAACTCC‑TACAGATGACCAGCCTTCCGCTGAGACAATCGAAGGTGTCACTAAGCCTTCCCACTACATGCTGTTTGACGACATTGAGGCTATCGAAGTGATTGCTCGTTCAATGACCGTTGAGCAGTTCAAGGGATACTGCTTCGGTAACATCTTAAAGTACAGACTAc                                                                                                                                                                              >  1:90451/1‑301 (MQ=255)`  
                                                                                                                                   `taagagacaggCGGTATTCTCTGCTCCAATGTCCCTTGCCACCTCTGCCCCGCAAATAACGATCAAAAGATAACCTTAGGTGAAATCCGAGCGATGGACCCACGTAAACCACATCTGAATAAACCTGAGGTAACTCC‑TACAGATGACCAGCCTTCCGCTGAGACAATCGAAGGTGTCACTAAGCCTTCCCACTACATGCTGTTTGACGACATTGAGGCTATCGAAGTGATTGCTCGTTCAATGACCGTTGAGCAGTTCAAGGGATACTGCTTCGGTAACATCTTAAAGTACAGACTACGTg                                                                                                                                                                           <  1:301005/291‑1 (MQ=255)`  
                                                                                                                                           `cTGCGGTATTCTCTGCTCCAATGTCCCTTGCCACCTCTGCCCCGCAAATAACGATCAAAAGATAACCTTAGGTGAAATCCGAGCGATGGACCCACGTAAACCACATCTGAATAAACCTGAGGTAACTCC‑TACAGATGATCAGCCTTCCGCTGAGACAATCGAAGGTGTCACTAAGCCTTCCCACTACATGCTGTTTGACGACATTGAGGCTATCGAAGTGATTGCTCGTTCAATGACCGTTGAGCAGTTCAAGGGATACTGCTTCGGTAACATCTTAAAGTACAGACTACGTGCTGGTaag                                                                                                                                                                   <  1:114761/301‑1 (MQ=255)`  
                                                                                                                                            `tgtgtataagagacagccAATGTCCCTTGCCACCTCTGCCCCGCAAATAACGATCAAAAGATAACCTTAGGTGAAATCCGAGCGATGGACCCACGTAAACCACATCTGAATAAACCTGAGGTAACTCC‑TACAGATGACCAGCCTTCCGCTGAGACAATCGAAGGTGTCACTAAGCCTTCCCACTACATGCTGTTTGACGACATTGAGGCTATCGAAGTGATTGCTCGTTCAATGACCGTTGAGCAGTTCAAGGGATACTGCTTCGGTAACATCTTAAAGTACAGACTACGTGCTGGTaaga                                                                                                                                                                  <  1:180961/285‑1 (MQ=255)`  
                                                                                                                                                `gTATTCTCTGCTCCAATGTCCCTTGCCACCTCTGCCCCGCAAATAACGATCAAAAGATAACCTTAGGTGAAATCCGAGCGATGGACCCACGTAAACCACATCTGAATAAACCTGAGGTAACTCC‑TACAGATGACCAGCCTTCCGCTGAGACAATCGAAGGTGTCACTAAGCCTTCCCACTACATGCTGTTTGACGACATTGAGGCTATCGAAGTGATTGCTCGTTCAATGACCGTTGAGCAGTTCAAGGGATACTGCTTCGGTAACATCTTaacctgtctcttatacacatctccgagccc                                                                                                                                                              >  1:331414/1‑273 (MQ=255)`  
                                                                                                                                                `gTATTCTCTGCTCCAATGTCCCTTGCCACCTCTGCCCCGCAAATAACGATCAAAAGATAACCTTAGGTGAAATCCGAGCGATGGACCCACGTAAACCACATCTGAATAAACCTGAGGTAACTCC‑TACAGATGACCAGCCTTCCGCTGAGACAATCGAAGGTGTCACTAAGCCTTCCCACTACATGCTGTTTGACGACATTGAGGCTATCGAAGTGATTGCTCGTTCAATGACCGTTGAGCAGTTCAAGGGATACTGCTTCGGTAACATCTTAAAGTACAGACTACGTGCTGGTAAGAAGTc                                                                                                                                                              >  1:205760/1‑301 (MQ=255)`  
                                                                                                                                                    `tctcTGCTCCAATGTCCCTTGCCACCCCTGCCCCGCAAATAACGATCAAAAGATAACCTTAGGTGAAATCCGAGCGATGGACCCACGTAAACCACATCTGAATAAACCTGAGGTAACTCC‑TACAGATGACCAGCCTTCCGCTGAGACAATCGAAGGTGTCACTAAGCCTTCCCACTACATGCTGTTTGACGACATTGAGGCTATCGAAGTGATTGCTCGTTCAATGACCGTTGAGCAGTTCAAGGGATACTGCTTCGGTAACATCTTAAAGTACAGACTACGTGCTGGTAAGAAGTCagag                                                                                                                                                          <  1:234100/301‑1 (MQ=255)`  
                                                                                                                                                       `ctGCTCCAATGTCCCTTGCCACCTCTGCCCCGCAAATAACGATCAAAAGATAACCTTAGGTGAAATCCGAGCGATGGACCCACGTAAACCACATCTGAATAAACCTGAGGTAACTCC‑TACAGATGACCAGCCTTCCGCTGAGACAATCGAAGGTGTCACTAAGCCTTCCCACTACATGCTGTTTGACGACATTGAGGCTATCGAAGTGATTGCTCGTTCAATGACCGTTGAGCAGTTCAAGGGATACTGCTTCGGTAACATCTTAAAGTACAGACTACGTGCTGGTAAGAAGTCAGAGTTa                                                                                                                                                       >  1:126054/1‑301 (MQ=255)`  
                                                                                                                                                              `aaTGTCCCTTGCCACCTCTGCCCCGCAAATAACGATCAAAAGATAACCTTAGGTGAACTCCGAGCGATGGACCCACGTAAACCACATCTGAATAAACCTGAGGTAACTCC‑TACAGATGACCAGCCTTCCGCTGAGACAATCGAAGGTGTCACTAAGACTTCCCACTACATGCTGTTTGACGACATTGAGGCTATCGAAGTGATTGCTCGTTCAATGACCATTGAGAAGTACAAGGGCTACTGCTTCGGTAACATCTTAAAGTACAAACTACGTGCTGGTACGAAGTCAGACttctttcact                                                                                                                                                >  1:338323/1‑293 (MQ=255)`  
                                                                                                                                                                 `gTCCCTTGCCACCTCTGCCCCGCAAATAACGATCAAAAGATAACCTTAGGTGAAATCCGAGCGATGGACCCACGTAAACCACATCTGAATAAACCTGATGTAACTCC‑TACAGATGACCAGCCTTCCGCTGAGACAATCGAAGGTGTCACTAAGCCTTCCCACTACATGCTGTTTGACGACATTGAGGCTATCGAAGTGATTGCTCGTTCAATGACCGTTGAGCAGTTCAAGGGATACTGCTTCGGTAACATCTTAAAGTACAGACTACGTGCTGGTAAGAAGTCAGAGTTAGCGTACTTaa                                                                                                                                             >  1:305021/1‑300 (MQ=255)`  
                                                                                                                                                                   `cccTTACCACCTCTGCCCCGCAAATAACGATCAAAAGATAACCTTAGGTGAAATCCGAGCGATGGACCCACGTAAACCACATCTGAATAAACCTGAGGTAACTCC‑TACAGATGACCAGCCTTCCGCTGAGACAATCGAAGGTGTCAATAAGCCTTCCCACTACATGCTGTTTGACGACATTGAGGCTATCGAAGTGATTGCTCGTTCAATGACCGTTGAGCAGTTCAAGGGATACTGCTTCGGTAACATCTTAAAGTACAGACTACGTGCTGTTAAGAAGTCAGAGTTAGCGTACTTagag                                                                                                                                           >  1:228170/1‑301 (MQ=255)`  
                                                                                                                                                                    `ccTTGCCACCTCTGCCCCGCAAATAACGATCAAAAGATAACCGTAGGTGAAATCCGAGCGATGGACCCACGTAAACCACATCTGAATAAACCTGAGGTAACTCC‑TACAGATGACCAGCCTTCCGCTGAGACAATCGAAGGTGTCACTAAGCCTTCCCACTACATGCTGTTTGACGACATTGAGGCTATCGAAGTGATTGCTCGTTCAATGACCGTTGAGCAGTTCAAGGGATACTGCTTCGGTAACATCTTAAAGTACAGACTACGTGCTGGTAAGAAGTCAGAGTTAGCGTACTTAgaga                                                                                                                                          <  1:263386/301‑1 (MQ=255)`  
                                                                                                                                                                    `ccTTGCCACCGCGGCCCCGAAAATAACGATCAAAAGAGAACCGTAGGTGAAATCCGAGCGATGGACCCACGTAAACCACATATGAATAAACCTGAGGTAACTCC‑GACAGATGACCAGCCTTCCGCTGAGACAATCGAAGGTGTCACTAAGCCTTCCCACTACATGCTGTTTGACGACATTGAGGCTATCGAAGTGATTGCTCGTTCAATGACCGTTGAGAAGTTCAAGGGATACTGCTTCGGTAACATCTTAAAGTACAGACTACGTGCTGGTAAGAAGTCAGAGTTAGAGTACTTAgaga                                                                                                                                          <  1:153869/301‑1 (MQ=255)`  
                                                                                                                                                                       `tGCCACCTCTGCCCCGCAAATAACGATCAAAAGATAACCTTAGGTGAAATCCGAGCGATGGACCCACGTAAACCACATCTGAATAAACCTGAGGTAACTCC‑TACAGATGACCAGCCTTCCGCTGAGACAATCGAAGGTGTCACTAAGCCTTCCCACTACATGCTGTTTGACGACATTGAGGCTATCGAAGTGATTGCTCGTTCAATGACCGTTGAGCAGTTCAAGGGATACTGCTTCGGTAACATCTTAAAGTACAGACTACGTGCTGGTAAGAAGTCAGCGTTAGCGTACTTAGAGaaaa                                                                                                                                       >  1:275182/1‑300 (MQ=255)`  
                                                                                                                                                                            `ccTCTGCCCCGCAAATAACGATCAAAAGATAACCTTAGGTGAAATCCGAGCGATGGACCCACGTAAACCACATCTGAATAAACCTGAGGTAACTCC‑TACAGATGACCAGCCTTCCGCTGAGACAATCGAAGGTGTCACTAAGCCTTCCCACTACATGCTGTTTGACGACATTGAGGCTATCGAAGTGATTGCTCGTTCAATGACCGTTGAGCAGTTCAAGGGATACTGCTTCGGTAACATCTTAAAGTACAGACTACGTGCTGGTAAGAAGTCAGAGTTAGCGTACTTAGAGAAAGACCTa                                                                                                                                  >  1:298157/1‑301 (MQ=255)`  
                                                                                                                                                                                      `tgtataagagacagaaaGATAACCTTAGGTGAAATCCGAGCGATGGACCCACGTAAACCACATCTGAATAAACCTGAGGTAACTCC‑TACAGATGACCAGCCTTCCGCTGAGACAATCGAAGGTGTCACTAAGCCTTCCCACTACATGCTGTTTGACGACATTGAGGCTATCGAAGTGATTGCTCGTTCAATGACCGTTGAGCAGTTCAAGGGATACTGCTTCGGTAACATCTTAAAGTACAGACTACGTGCTGGTAAGAAGTCAGAGTTAGCGTACTTAGAGAAAGACCTAGCGAAAGCAg                                                                                                                        <  1:65761/287‑1 (MQ=255)`  
                                                                                                                                                                                      `gCAAATAACGATCAAAAGATAACCTTAGGTGAAATCCGAGCGATGGACCCACGTAAACCACATCTGAATAAACCTGAGGTAACTCC‑TACAGATGACCAGCCTTCCGCTGAGACAATCGAAGGTGTCACTAAGCCTTCCCACTACATGCTGTTTGACGACATTGAGGCTATCGAAGTGATTGCTCGTTCAATGACCGTTGAGCAGTTCAAGGGATACTGCTTCGGTAACATCTTAAAGTACAGACTACGTGCTGGTAAGAAGTCAGAGTTAGCGTACTTAGAGAAAGACCTAGCGAAAGCAg                                                                                                                        <  1:246505/301‑1 (MQ=255)`  
                                                                                                                                                                                        `aaaTAACGAGCAAAAGATAACCTTAGGTGAAATCCGAGCGATGGACCCACGTAAACCACATCTGAATAAACCTGAGGTAACTCC‑TACAGATGACCAGCCTTCCGCTGAGACAATCGAAGGTGTCACTAAACCTTCCCACTACATGCTGTTTGACGACATTGAGGCTATCGAAGTGATTGCTCGTTCAATGACCGTTGAGCAGTTCAAGGGATACTGCTTCGGTAACATCTTAAAGTACAGACTACGTGCTGGTAAGAAGTCAGAGTTAGCGTACTTAGAGAAAGACCTAGCGAAAGCAGAc                                                                                                                      <  1:135966/301‑1 (MQ=255)`  
                                                                                                                                                                                                `aTCAAAAGATACCCTTAGGGGAAATCCGAGCGATTGACCCACGTAAACCACATCTGAATCAACCTGAGGTAACTCC‑TACAGATGACCAACCTTCCGCTGAGACAATCGAAGGTGTCACTAAGCCTTCCCACTGCATGCTGTTTTACGACAGTGATGCTATCGAAGTGATTGCTCGTTCAATGACCGTTGAGCAGTTCAAGGGATACTGCTTCGGTAACATCTTAAAGTACAGACTACGTGCTGGTAAGAAGTCAGAGTTAGCGTACTTAGAGAAAGACCTAGCGAAAGCAGACTTCTATaa                                                                                                              <  1:313161/301‑1 (MQ=255)`  
                                                                                                                                                                                                     `gagacagccTTAGGTGAAATCCGAGCGATGGACCCACGTAAACCACATCTGAATAAACCTGAGGTAACTCC‑TACAGATGACCAGCCTTCCGCTGAGACAATCGAAGGTGTCACTAAGCCTTCCCACTACATGCTGTTTGACGACATTGAGGCTATCGAAGTGATTGCTCGTTCAATGACCGTTGAGCAGTTCAAGGGATACTGCTTCGGTAACATCTTAAAGTACAGACTACGTGCTGGTAAGAAGTCAGAGTTAGCGTACTTAGAGAAAGACCTAGCGAAAGCAGACTTCTATAAAGAAc                                                                                                         <  1:22252/294‑1 (MQ=255)`  
                                                                                                                                                                                                     `atgtatataagagacagaaTCTGAGCGAGTGACCCACGTAAACCACATATGAATAAACCTGAGGTAACTCC‑TACAGATGACCAGCCTTCCGCTGAGACAATCGAAGGTGTCACTAAGCCTTCCCACTACATGCTGTTTGACGACATTGAGGCTATCGAAGTGATTGCTCGTTCAATGACCGTTGAGCAGTTCAAGGGATACTGCTTCGGTAACATCTTAAAGTACAGACTACGTGCTGGTAAGAAGTCAGAGTTAGCGTACTTAGAGAAAGACCTAGCGAAAGCAGACTTCAATAAAGAAc                                                                                                         <  1:45916/284‑1 (MQ=255)`  
                                                                                                                                                                                                      `aGATAACCTTAGGTGAAATCCGAGCGATGGACCCACGTAAACCACATCTGAATAAACCTGAGGTAACTCC‑TACAGATGACCAGCCTTCCGCTGAGACAATCGAAGGTGTCACTAAGCCTTCCCACTACATGCTGTTTGACGACATTGAGGCTATCGAAGTGATTGCTCGTTCAATGACCGTTGAGCAGTTCAAGGGATACTGCTTCGGTAACATCTTAAAGTACAGACTACGTGCTGGTAAGAAGTCAGAGTTAGCGTACTTAGAGAAAGACCTAGCgctgtctcttatacacatctccga                                                                                                        >  1:243287/1‑278 (MQ=255)`  
                                                                                                                                                                                                      `aGATAACCTTAGGTGAAATCCGAGCGATGGACCCACGTAAACCACATCTGAATAAACCTGAGGTAACTCC‑TACAGATGACCAGCCTTCCGCTGAGACAATCGAAGGTGTCACTAAGCCTTCCCACTACATGCTGTTTAACGACATTGAGGCTATCGAAGTGATTGCTCGTTCAATGACCGTTGAGCAGTTCAAGGGATACTGCTTCGGTAACATCTTAAAGTACAGACTACGTGCTGGTAAGAAGTCAGAGTTAGCGTACTTAGAGAAAGACCTAGCGAAAGCAGACTTCTATAAAGAAct                                                                                                        >  1:141494/1‑301 (MQ=255)`  
                                                                                                                                                                                                             `cTTAGGTGAAATCCTAGCGATGGACCCACGTAAACCACATCTGAATAAACCTGAGGTAACTCC‑TACAGATGACCAGCCTTCCGCTGAGACAATCGAAGGTGTCACTAAGCCTTCCCACTACATGCTGTTTGACGACATTGAGGCTATCGAAGTGATTGCTCGTTCAATGACCGTTGAGCAGTTCAAGGGATACTGCTTCGGTAACATCTTAAAGTACAGACTACGTGCTGGAAAGAAGTCAGAGTTAGCGTACTTAGAGAAAGACCTAGCGAAAGCAGACTTCTATAAAGAACTCTTTgag                                                                                                 <  1:163697/301‑1 (MQ=255)`  
                                                                                                                                                                                                                 `ggTGAAATCCGAGCGATGGACCCACGTAAACCACATCTGAATAAACCTGAGGTAACTCC‑TACAGATGACCAGCCTTCCGCTGAGACAATCGAAGGTGTCACTAAGCCTTACCACTACATGCTGTTTGACGACATTGAGGCTATCGAAGTGATTGCTCGTTCAATGACCGTTGAGCAGTTCAAGGGATACTGCTTCGGTAACATCTTAAAGTACAGACTACGTGCTGGTAAGAAGTAAGAGTTAGCGTACTTAGAGAAAGACCTAGCGAACGCAGACTTCtatatgtctgttatacccatct                                                                                             >  1:166308/1‑283 (MQ=255)`  
                                                                                                                                                                                                                        `ggagatgtgtataagagacagaaCCACATCTGAATAAACCTGAGGTAACTCC‑TACAGATGACCAACCTTCCGCTGAGACAATCGAAGGTGTCACTAAGCCTTCCCACTACATGCTGTTTGACGACATTGAGGCTATCGAAGTGATTGCTCGTTCAATGACCGTTGAGCAGTTCAAGGGATACTGCTTCGGTAACATCTTAAAGTACAGACTACGTGCTGGTAAGAAGTCAGAGTTAGCGTACTTAGAGAAAGACCTAGCGAAAGCAGACTTCTATAAAGAACTCTTTGAGAAACATAAGGa                                                                                      <  1:186712/280‑1 (MQ=255)`  
                                                                                                                                                                                                                              `ggATGTACCCACGTAAACCACATCTGAATACACCTGAGGTAACTCC‑TACAGATGACCAGCCTTCCGCTGAGACAATCGAAGGTGTCACTAAGCCTTCCCACTACATGCTGTTTGACGACATTGAGTCTATCGAAGTGATTGCTCGTTCAATGACCGTTGAGCAGTTCAAGGGATACTGCTTCGGTAACATCTTAAAGTACAGACTACGTGCTGGTAAGAAGTCAGAGTTAGCGTACTTAGAGAAAGACCTAGCGAAAGCAGACTTCTATAAAGAACTCTTTGAGAAACATAAGGATAAATg                                                                                <  1:340516/300‑1 (MQ=255)`  
                                                                                                                                                                                                                               `aagggactaacctAAACCACATCTTAAGAAACCTGAGGTAACTCCTTACA‑AAGACCAGCCTTCCGCTGAGACAATCGAAGGTGTCACTAAGCCTTCCCACTACATGCTGTTTGACGACATTGAGGCTATCGAAGTGATTGCTCGTTCGATGACCGTTGAGCAGTTCAAGGGAAACTGCTTCGGTAACATCTTAAAGTACAGACTACGTGCTAGTAAGAAGTCAGAGTTAGCGTACTTAGAGAAAGACCTAGCGAAAGCAGACTTCTATAAAGAACTCTTTGAGAAACATAAGGATAAATGt                                                                               <  1:199831/289‑1 (MQ=255)`  
                                                                                                                                                                                                                                    `aCCCACGTAAACCACATCTGAATAAACCTGAGGTAACTCC‑TACAGATGACCAGCCTTCCGCTGAGACAATCGAAGGTGTCACTAAGCCTTCCCACTACATGCTGTTTGACGACATTGAGGCTATCGAAGTGATTGCTCGTTCAATGACCGTTGAGCAGTTCAAGGGATACTGCTTCGGTAACATCTTAAAGTACAGACTACGTGCTGGTAAGAAGTCAGAGTTAGCGTACTTAGAGAAAGACCTAGCGAAAGCAGACTTCTATAAAGAACTCTTTGAGAAACATAAGGATAAATGTTATGc                                                                          <  1:100290/301‑1 (MQ=255)`  
                                                                                                                                                                                                                                      `gggctcggagatgtgtataagagacaggAGGTAACTCC‑TACAGATGACCAGCCTTCCGCTGAGACAATCGAAGGTGTCACTAAGCCTTCCCACTACATGCTGTTTGACGACATTGAGGCTATCGAAGTGATTGCTCGTTCAATGACCGTTGAGCAGTTCAAGGGATACTGCTTCGGTAACATCTTAAAGTACAGACTACGTGCTGGTAAGAAGTCAGAGTTAGCGTACTTAGAGAAAGACCTAGCGAAAGAAGACTTCTATAAAGAACTCTTTGAGCAACATAAGGATAAATGTTATGCAt                                                                        <  1:294914/274‑1 (MQ=255)`  
                                                                                                                                                                                                                                      `ccACGTAAACCACATCTGAATAAACCTGAGGTAACTCC‑TACAGATGACCAGCCTTCCACTGAGACAATCGAAGGTGTCACTAAGCCTTCCCACTACATGCTGTTTGACGACATTGAGGCTATCGAAGTGATTGCTCGTTCAATGACCGTTGAGCAGTTCAAGGGATACTGCTTCGGTAACATCTTAAAGTACAGACTACGTGCTGGTAAGAAGTCAGAGTTAGCGTACTTAGAGAAAGACCTAGCGAAAGCAGACTTCTATAAAGAACTCTTTGAGAAACATAAGGATAAATGTTATGCAt                                                                        <  1:253392/301‑1 (MQ=255)`  
                                                                                                                                                                                                                                         `cGTAAACCACATCTGAAGAAACCTGAGGTAACTCC‑TACAGAGGACCAGCCTTCCGCTGAGACAATCGAAGGTGTCACTAAGCCTTCCCACTACATGCTGTTTGACGACATTGAGGCTATCGAAGTGATTGTTCGTTCAATGACCGTTGAGCAGTTCAAGGGATACTGCTTCGGTAACATCTTAAAGTACAGACTACGTGCTGGTAAGAAGTCAGAGTTAGCGTACTTAGAGAAAGACCTAGCGAAAGCAGACTTCTATAAAGAACTCTTTGAGAAACATAAGGATAAATGTTATGCATAAc                                                                     <  1:84765/301‑1 (MQ=255)`  
                                                                                                                                                                                                                                            `aaaCCACATCTGAATAAACCTTAGTTAACTCC‑TACAGATGACCCGCCTTCCGCTGAGACAATCGAAGGTGTCACTAAGCCTTCCCACTACATGCTGTTTGACGACATTGAGTCTATCGAAGTTATTGCTCGTTCAATGACCGTTGAGCAGTTCAAGGGATACTGCTTCGGTAACATCTTAAAGTACAGACTACGTGCTGGTAAGAAGTCAGAGTTAGCGTACTTAGAGAAAGACCTAGCGAAAGCAGACTTCTATAAAGAACTCTTTGAGAAACATAAGGATAAATGTTATGCATAACTTc                                                                  <  1:103128/301‑1 (MQ=255)`  
                                                                                                                                                                                                                                            `aaaCCACAGCTGAATAAACCTGAGTTAACTCC‑TACAGATTACCAGCCTTCCGCTGAGACAATCGAAGGGGTCACTAAGCCTTCCCACTACATGCTGTTTGACGACATTGAGGCTATCGAAGTGATTGCTCGTTCAATGACCGTTGAGCAGTTCAAGGGATACTGCTTCGGTAACATCTTAAAGTACAGACTACGTGCTGGTAAGAAGTCAGAGTTAGCGTACTTAGAGAAAGACCTAGCGAAAGCAGACTTCTATAAAGAACTCTTTGAGAAACATAAGGATAAATGTTATGCATAACTTc                                                                  <  1:328122/301‑1 (MQ=255)`  
                                                                                                                                                                                                                                                 `agacagaaaTAAACATGAGGGAACTCC‑TACAGATGACCAGCCTTCCGCTGAGACAATAGAAGGGGTCACTAAGCCTTCCCACTACATGCTGTTTGACGACATGGAGGCTATCGAAGTGATTGCTCGTTCAATGACCGTTGAGCAGTTCAAGGGATACTGCTTCGGTAACATCTTAAAGTACAGACTACGTGCTGGTAAGAAGTCAGAGTTAGCGTACTTAGAGAAAGACCTAGCGAAAGCAGACTTCTATAAAGAACTCTTTGAGAAACATAAGGATAAATGTTATGCATAACTTCAAGTc                                                             <  1:209751/294‑1 (MQ=255)`  
                                                                                                                                                                                                                                                       `gAATAAACCTGAGGTAACTCC‑TACAGATGACCAGCCTTCCGCTGAGACAATCGAAGGTGTCACTAAGCCTTCCCACTACATGCTGTTTGACGACATTGAGGCTATCGAAGTGATTGCTCGTTCAATGTCCGTTTAGCAGTTCAAGGGCTACTGCTTCGGTCACATCTTAAAGTATAGACTACGTGCTGGTAAGAAGTAAGAGTTAGCGTAATTAGAGAAAGACCTTGCGAAAGCAGAATTCTATAAAGAACTCTTTGAGAAACATAAGGATAAAGGTTATGAATatgtctcttatacacat                                                       >  1:183994/1‑285 (MQ=255)`  
                                                                                                                                                                                                                                                       `gAATAAACATGAGGTAACTCC‑TACAGATGACCAGCCTTCCGCTGAGACAATCGAAGGTGTCACTAAGCCTTCCCACTACATGCTGTTTGACGACATTGAGGCTATCGAAGTGATTGCTCATTAAATGACCGTTGAGCAGTACAAGGGATACTGCTTAGATAAAATCTTAAAGTACAGACTAAGTGCTGGTAAGAAGTCAGAGTTAGCGTACTTAGAGAAAGACCTAGCGAAAGCAGACTTCTATCAAGAAATCTTTACGAAACATAAGAAGAAATGTAATGAAAAACTTAAAATCAAccca                                                       >  1:36803/1‑300 (MQ=255)`  
                                                                                                                                                                                                                                                            `aaCCTGAGGTAACTCC‑TACAGATGACCAGCCTTCCGCTGAGACAATCGAAGGTGTCACTAAGCCTTCCCACTACATTCTGTTTGACGACATTGCGGCTATCGAAGTGATTGCTCTTTCAATGACCGTTGAGCAGTTCAAGGGATACTGCTTCGGTAACATCTTAAAGTACAGACTACGTGCTGGTAAGAAGTCAGAGTTAGCGTACTTAGAGAAAGACCTAGCGAAAGCAGACTTCTATAAAGAACTCTTTGAGAAACATAAGGATAAATGTTATGCATACCTTCAAGTCAACCCCACCTg                                                  >  1:57603/1‑301 (MQ=255)`  
                                                                                                                                                                                                                                                              `ccTGAGGTAACTCC‑TACAGATGACCAGCCTTCCGCTGAGACAATCGAAGGTGTCACTAAGCCTTCCCACTACATGCTGTTTGACGACATTGAGGCTATCGAAGTGATTGCTCGTTCAATGACCGTTGAGCAGTTCAAGGGATACTGCTTCGGTAACATCTTAAAGTACAGACTACGTGCTGGTAAGAAGTCAGAGTTAGCGTACTTAGAGAAAGACCTAGCGAAAGCAGACTTCTATAAAGAACTCTTTGAGAAACATAAGGATAAATGTTATGcctgtctcttatacacatctccgagcc                                                >  1:270238/1‑275 (MQ=255)`  
                                                                                                                                                                                                                                                                 `gAGGTAACTCC‑TACAGATGACCAGCCTTCCGCTGAGACAATCGAAGGTGTCACTAAGCCTTCCCACTACATGCTGTTTGACGACATTGAGGCTATCGAAGTGATTGCTCGTTCAATGACCGTTGAGCAGTTCAAGGGATACTGCTTCGGTAACATCTTAAAGTACAGACTACGTGCTGGTAAGAAGTCAGAGTTAGCGTACTTAGAGAAAGACCTAGCGAAAGCAGACTTCTATAAAGAACTCTTTGAGAAACATAAGGATAAATGTTATGCATAACTTCAAGTCAACCCCACCTGCCGAc                                             >  1:265399/1‑301 (MQ=255)`  
                                                                                                                                                                                                                                                                      `aaCTCC‑TACAGATGACCAGCCTTCCGCTGAGACAATCGAAGGTGTCACTAAGCCTTCCCACTACATGCTGTTTGACGACATTGAGGCTATCGAAGTGATTGCTCGTTCAATGACCGTTGAGCAGTTCAAGGGATACTGCTTCGGTAACATCTTAAAGTACAGACTACGTGCTGGTAAGAAGTCAGAGTTAGCGTACTTAGAGAAAGACCTAGCGAAAGCAGACTTCTATAAAGAACTCTTTGAGAAACATAAGGATAAATGTTATGCATAACTTCAAGTCAACCCCACCTGCCGACAGCCt                                        >  1:78373/1‑301 (MQ=255)`  
                                                                                                                                                                                                                                                                        `cTCC‑TACAGATGACCAGCCTTCCGCTGAGACAATCGAAGGTGTCACTAAGCCTTCCCACTACATGCTGTTTGACGACATTGAGGCTATCGAAGTGATTGCTCGTTCAATGACCGTTGAGCAGTTCAAGGGATACTGCTTCGGTAACATCTTAAAGTACAGACTACGTGCTGGTAAGAAGTCAGAGTTAGCGTACTTAGAGAAAGACCTAGCGAAAGCAGACTTCTATAAAGAACTCTTTGAGAAACATAAGGATAAATGTTATGCATAACTTCAAGTCAACCCCACCTGCCGACAGCCTAt                                      >  1:277386/1‑301 (MQ=255)`  
                                                                                                                                                                                                                                                                             `tACAGATGACCAGCCTTCCGCTGAGACAATCGAAGGTGTCACTAAGCCTTCCCACTACATGCTGTTTGACGACATTGAGGCTATCGAAGTGATTGCTCGTTCAATGACCGTTGAGCAGTTCAAGGGATACTGCTTCGGTAACATCTTAAAGTACAGACTACGTGCTGGTAAGAAGTCAGAGTTAGCGTACTTAGAGAAAGACCTAGCGAAAGCAGACTTCTATAAAGAACTCTTTGAGAAACATAAGGATAAATGTTATGCATAACTTCAAGTCAACCCCACCTGCCGACAGCCTATCtga                                  <  1:308785/301‑1 (MQ=255)`  
                                                                                                                                                                                                                                                                             `tACAGATGACCAGCCTTCCGCTGAGACAATCGAAGGTGTCACTAAGCCTTCCCACTACATGCTGTTTGACGACATTGAGGCTATCGAAGTGATTGCTCGTTCAATGACCGTTGAGCAGTTCAAGGGATACTGCTTCGGTAACATCTTAAAGTACAGACTACGTGCTGGTAAGAAGTCAGAGTTAGCGTACTTAGAGAAAGACCTAGCGAAAGCAGACTTCTATAAAGAACTCTTTGAGAAACATAAGGATAAATGTTATGCATAACTTCAAGTCAACCCCACCTGCCGACAGCCTATCtga                                  <  1:193632/301‑1 (MQ=255)`  
                                                                                                                                                                                                                                                                                    `gACCAGCCGTCCGCTGAGGAAATCGAAGGTGTCACTAAGCCTTCCCACTACATGCTGTTTGACGACATTGAGGCTATCGAAGTGATTGCTCGTTCAATGACCGTTGAGCAGTTCAAGGGATACTGCTTCGGTAACATCTTAAAGTACAGACTACGTGCTGGTAAGAAGTCAGAGTTAGCGTACTTAGAGAAAGACCTAGCGAAAGCAGACTTCTATAAAGAACTCTTTGAGAAACATAAGGATAAATGTTATGCATAACTTCAAGTCAACCCCACCTGCCGACAGCCTATCTGATGACTTc                           <  1:344105/301‑1 (MQ=255)`  
                                                                                                                                                                                                                                                                                    `gACCAGACTTCCGCTGAGACAATCGAAGGTGTCACTAAGCCTTCCCACTACATGCTGTTTGACGACATTGAGGCTAGCGAAGTGAGTGCTCGTTCAATGACCGTTGAGCAGTTCAAGGGATACTGCTTCGGTAACATCTTAAAGTACAGACTACGTGCTGGTAAGAAGTCAGAGTTAGCGTACTTAGAGAAAGACCTAGCGAAAGCAGACTTCTATAAAGAACTCTTTGAGAAACATAAGGATAAATGTTATGCATAACTTCAAGTCAACCCCACCTGCCGACAGCCTATCTGATGACTTc                           <  1:324240/301‑1 (MQ=255)`  
                                                                                                                                                                                                                                                                                               `cGCTGAGACAATCGAAGGTGTCACTAAGCCTTCCCACTACATGCTGTTTGACGACATTGAGGCTATCGAAGTGATTGCTCGTTCAATGACCGTTGAGCAGTTCAAGGGATACTGCTTCGGTAACATCTTAAAGTACAGACTACGTGCTGGTAAGAAGTCAGAGTTAGCGTACTTAGAGAAAGACCTAGCGAAAGCAGACTTCTATAAAGAACTCTTTGAGAAACATAAGGATAAATGTTATGCATAACTTCAAGTCAACCCCACCTGCCGACAGCCTATCTGATGACTTCACATCTTGCTc                <  1:202868/301‑1 (MQ=255)`  
                                                                                                                                                                                                                                                                                                    `agaCAATCGAAGGTGTCACTAAGCCTTCCCACTACATGCTGTTTGACGACATTGAGGCTATCGAAGTGATTGCTCGTTCAATGACTGTTGAGCAGTTCAAGGGATACTGCTTCGGTAACATCTTAAAGTACAGACTACGTTCTGGTAAGAAGTCAGAGTAAGCGTACTTAGAGAAAGACCTAGCGAAAGCAGACTTCTATAAAGAACTCTTTGAGAAACATAAGGATAAATGTTATGCATAACTTCAAGTCAACCCCACCTGCCGACAGCCTATCTGATGACTTCACATCTTGCTCAGAgt           <  1:61489/301‑1 (MQ=255)`  
                                                                                                                                                                                                                                                                                                       `cAATCGAAGGTGTCACTAAGCCTTCCCACTACATGCTGTTTGACGACATTGAGGCTATCGAAGTGATTGCTCGTTCAATGACCGTTGAGCAGTTCAAGGGATACTGCTTCGGTAACATCTTAAAGTACAGACTACGTGCTGGTAAGAAGTCAGAGTTAGCGTACTTAGAGAAAGACCTAGCGAAAGCAGACTTCTATAAAGAACTCTTTGAGAAACATAAGGATAAATGTTATGCATAACTTCAAGTCAACCCCACCTGCCGACAGCCTATCTGATGACTTcctgtctcttatacacatct        >  1:81136/1‑282 (MQ=255)`  

                                                                                                                                                                                                                                                                                                             `|`                                                                                                                                                                                                                                                                                                              
`GGTGTATAGCTTGCTTAGAGAAAGATGACCGTTATCCACACACCTGTAACAAAGGAGCTAACGATATGACCGAACGTGAACAAGAGATGATCATTAAGTTGATAGACAATAATGAAGGTCGCCCAGATGATTTGAATGGCTGCGGTATTCTCTGCTCCAATGTCCCTTGCCACCTCTGCCCCGCAAATAACGATCAAAAGATAACCTTAGGTGAAATCCGAGCGATGGACCCACGTAAACCACATCTGAATAAACCTGAGGTAACTCC‑TACAGATGACCAGCCTTCCGCTGAGACAATCGAAGGTGTCACTAAGCCTTCCCACTACATGCTGTTTGACGACATTGAGGCTATCGAAGTGATTGCTCGTTCAATGACCGTTGAGCAGTTCAAGGGATACTGCTTCGGTAACATCTTAAAGTACAGACTACGTGCTGGTAAGAAGTCAGAGTTAGCGTACTTAGAGAAAGACCTAGCGAAAGCAGACTTCTATAAAGAACTCTTTGAGAAACATAAGGATAAATGTTATGCATAACTTCAAGTCAACCCCACCTGCCGACAGCCTATCTGATGACTTCACATCTTGCTCAGAGTGGTGCCGAA  >  NC_001604.1/8224‑8824`

  

Alignment Legend

Aligned base mismatch/match (shaded by quality score): `ATCG/ATCG` `< 3 ≤` `ATCG/ATCG` `< 11 ≤` `ATCG/ATCG` `< 24 ≤` `ATCG/ATCG` `< 36 ≤` `ATCG/ATCG` `< 38 ≤` `ATCG/ATCG`

Unaligned base: `atcg`    Masked matching base: `atcg`    Alignment gap: `‑`    Deleted base: `‑`

Reads not counted as support for junction

read\_name Not counted due to insufficient overlap past the breakpoint.

read\_name Not counted due to not crossing MOB target site duplication.
